# Tolerability and adverse events in clinical trials of celecoxib in osteoarthritis and rheumatoid arthritis: systematic review and meta-analysis of information from company clinical trial reports

**DOI:** 10.1186/ar1704

**Published:** 2005-03-24

**Authors:** R Andrew Moore, Sheena Derry, Geoffrey T Makinson, Henry J McQuay

**Affiliations:** 1 Pain Research and Nuffield Department of Anaesthetics, University of Oxford, Oxford Radcliffe NHS Trust, Oxford, UK; 2 Department of Outcomes Research and Evidence-based Medicine, Pfizer Ltd, Walton Oaks, Surrey, UK

## Abstract

The objective was to improve understanding of adverse events occurring with celecoxib in the treatment of osteoarthritis and rheumatoid arthritis. Data were extracted from company clinical trial reports of randomised trials of celecoxib in osteoarthritis or rheumatoid arthritis lasting 2 weeks or more. Outcomes were discontinuations (all cause, lack of efficacy, adverse event, gastrointestinal adverse event), endoscopically detected ulcers, gastrointestinal or cardio-renal events, and major changes in haematological parameters. The main comparisons were celecoxib (all doses) versus placebo, paracetamol (acetaminophen) 4,000 mg daily, rofecoxib 25 mg daily, or nonsteroidal anti-inflammatory drugs (NSAIDs) (naproxen, diclofenac, ibuprofen, and loxoprofen). For NSAIDs, celecoxib was compared both at all doses and at licensed doses (200 to 400 mg daily). Thirty-one trials included 39,605 randomised patients. Most patients had osteoarthritis and were women of average age 60 years or above. Most trials lasted 12 weeks or more. Doses of celecoxib were 50 to 800 mg/day. Compared with placebo, celecoxib had fewer discontinuations for any cause or for lack of efficacy, fewer serious adverse events, and less nausea. It had more patients with dyspepsia, diarrhoea, oedema, more adverse events that were gastrointestinal or treatment related, and more patients experiencing an adverse event. There were no differences for hypertension, gastrointestinal tolerability, or discontinuations for adverse events. Compared with paracetamol, celecoxib had fewer discontinuations for any cause, for lack of efficacy, or diarrhoea, but no other differences. Compared with rofecoxib, celecoxib had fewer patients with abdominal pain and oedema, but no other differences. Compared with NSAIDs, celecoxib had fewer symptomatic ulcers and bleeds, endoscopically detected ulcers, and discontinuations for adverse events or gastrointestinal adverse events. Fewer patients had any, or a gastrointestinal, or a treatment-related adverse event, or vomiting, abdominal pain, dyspepsia, or reduced haemoglobin or haematocrit. Discontinuations for lack of efficacy were higher. No differences were found for all-cause discontinuations, serious adverse events, hypertension, diarrhoea, nausea, oedema, myocardial infarction, cardiac failure, or raised creatinine. Company clinical trial reports present much more information than published papers. Adverse event information is clearly presented in company clinical trial reports, which are an ideal source of information for systematic review and meta-analysis.

## Introduction

Arthritis is a common, progressive condition, which is associated with considerable pain and inflammation, and has a strong impact on quality of life. It is the major reason for hip or knee replacements [1].

It is more prevalent in women than men, and in older people. One community-based study [2] conducted in Scotland showed that 25% of patients had arthritis by age 65. Of these, a quarter had pain that was highly disabling and at least moderately limiting. A further quarter had pain that was more severe. In a UK general practice survey of patients' perspectives in osteoarthritis [3], a quarter of responders reported some dissatisfaction with their treatment and another quarter stated that their pain control was poor. High levels of negative impact were associated with inability to walk, bathe, dress, or sleep, with 40% of patients saying that these activities were often or always affected. A quarter of patients used over-the-counter medicines, mainly paracetamol or ibuprofen, in addition to those prescribed by their doctor. Half of responders were over age 65, and two-thirds were women.

Drug treatment is ideally effective, safe, and well tolerated. NSAIDs have provided the mainstay of pain therapy, particularly in the early stages of disease, but are often associated with clinically relevant adverse events.

Common events such as nausea or dizziness, often considered minor, can have an impact on people's lives and reduce compliance with prescribed dose. Patients with arthritis avoid adverse events, choosing less effective medicine with less likelihood of adverse events over more effective medicine with more adverse events [4]. Only 20% of patients with arthritis prescribed NSAIDs will be taking the same drug after one year [5], adverse events being a major reason for discontinuation.

Serious adverse events occur infrequently, but the consequence to the individual may be considerable. With conventional NSAIDs, there is the risk of major harm through gastrointestinal ulceration, perforation, and bleeding. These events consume considerable resources through cost of hospitalisation and treatment, or through coprescription of gastroprotective agents to minimise the risk of major harm [6].

Cox-2-selective inhibitors (coxibs) are an alternative to NSAIDs, developed to give better gastrointestinal safety and tolerability. For evaluation of the adverse-event profiles of coxibs, outcomes of interest include endoscopically detected ulcers and erosions, and symptomatic ulcers, which may progress to bleeding ulcers, and can even cause death [7]. Renal failure [8,9] and heart failure [10,11] also occur with NSAIDs or coxibs. Other adverse event outcomes that are useful to know include those describing discontinuation (early withdrawal from the trial), particularly discontinuation because of adverse events or lack of efficacy.

This systematic review and meta-analysis of celecoxib in osteoarthritis and rheumatoid arthritis was conducted using information from company clinical trial reports, supplied by Pfizer Ltd, of completed randomised, double-blind trials from the celecoxib clinical trials programme. The objectives were to examine tolerability, minor and major adverse events, and endoscopically detected ulceration associated with celecoxib in arthritis.

## Materials and methods

Randomised, double-blind, controlled trials, of 2 weeks' duration or longer with any dose of celecoxib and any comparator, in osteoarthritis or rheumatoid arthritis, were supplied as company clinical trial reports by Pfizer Ltd. Open-label extension studies were not included. A declaration was signed by Pfizer that all completed (by December 2003) trials of relevance from the celecoxib clinical trial programme had been made available. A protocol for the review and analysis, including definitions of outcomes, was agreed beforehand.

Financial support was provided by Pfizer Ltd, with the provision that all relevant trial reports completed by December 2003 were made available, and that the authors were free to publish their findings whatever the outcome of the review. Other funding was from Pain Research funds of the Oxford Pain Relief Trust. No funding source had any role in deciding what to publish, when to publish, or where to publish it.

### Trials

Thirty-one Phase II, III, and IV clinical trial reports of celecoxib in osteoarthritis or rheumatoid arthritis were provided for evaluation. All compared celecoxib in various dosing regimens with placebo, paracetamol (acetaminophen) 4,000 mg/day, rofecoxib 25 mg/day, or an NSAID commonly used in the treatment of arthritis. Comparator NSAIDs were given at the maximum licensed dose; these were naproxen 1,000 mg, ibuprofen 2,400 mg, diclofenac 100 to 150 mg, and loxoprofen 180 mg daily. Details of the included trials are in Table 1.

#### Trial inclusion and exclusion criteria

Patients were adults who had a clinical diagnosis of osteoarthritis or rheumatoid arthritis that was symptomatic, usually of 3 months' duration or longer, and required long-term treatment with anti-inflammatory drugs or other analgesics for the control of pain. Further details of inclusion and exclusion criteria for both osteoarthritis and rheumatoid arthritis can be found in Additional file 1.

#### Trial methods

Eligible patients typically entered a pretreatment period of up to 14 days, during which baseline observations were conducted. Nonstudy NSAIDs and other analgesics were discontinued, with the exception of aspirin (up to 325 mg daily) and paracetamol (up to 2 g per day for a maximum of 3 days but not within 48 hours of arthritis assessments), which were permitted for reasons other than control of arthritis pain. Other drugs specifically excluded were antibiotics for *Helicobacter pylori *eradication, metronidazole, anticoagulants, lithium, and anti-ulcer drugs including proton pump inhibitors, H2 antagonists, antacids, sucralfate, and misoprostol.

Patients were randomised under double-blind conditions to receive oral celecoxib, paracetamol, rofecoxib, an NSAID, or placebo. Several studies had both an active and a placebo comparator, and several compared different fixed dose regimens of celecoxib. Table 1 shows the study treatments, dosing, and number and baseline characteristics of patients for the individual trials. All trials conformed to good clinical practice guidelines.

#### Information collected on adverse events

In all studies, information was collected on patients who experienced any adverse event, serious adverse events, adverse events relating to body systems, and discontinuations. Information was collected on the occurrence of endoscopically detected ulcers and erosions from those trials in which all patients were scheduled to have endoscopy before and at various times during treatment. Definitions used in the trials were those of the World Health Organization (Adverse Reaction Terminology). The definitions used in this review are in Additional file 2.

### Meta-analysis

#### Outcomes chosen for the meta-analysis

Outcomes chosen related to adverse events and tolerability. These included discontinuation (all-cause, lack of efficacy, adverse event, and gastrointestinal adverse event), patients with any adverse event, patients with any treatment-related adverse event, and patients with any serious adverse event.

For gastrointestinal adverse events, we included an overall measure of gastrointestinal tolerability as well as individual gastrointestinal adverse events of nausea, vomiting, abdominal pain, dyspepsia, diarrhoea, and ulcers or bleeds. Treatment-emergent ulcers and bleeds were analysed together because of their important sequelae. Endoscopically detected ulcers were taken from reports in which all patients in the trial had endoscopy with the specific intent of measuring endoscopic lesions, and where this was a prime outcome in the trial. They were additionally analysed according to the concomitant use of low-dose aspirin.

Specific cardio-renal adverse events included cardiac failure, hypertension, raised creatinine, and oedema at any body site. Analysis of oedema by body site, or hypertension by subcategory, was not carried out, as event numbers were too low for practicable analysis.

#### Trial quality and validity

Three authors independently read each clinical trial report and scored the reports for reporting quality and validity. Disagreements were discussed and consensus achieved. Trials were scored for quality using a three-item, 1- to 5-point scale [12], and at least two points, one each for randomisation and double blinding, were required for inclusion. Trials were scored for validity using an eight-item, 16-point scale [13]; there was no minimum requirement for inclusion in the systematic review.

#### Analysis

Guidelines for quality of reporting of meta-analyses were followed where appropriate [14].

The prior intention was to pool data where there was clinical homogeneity, with similarity in terms of patients, dose, duration, outcomes, and comparators. It was recognised, however, that this could lead to a large number of comparisons, with small numbers of events, where random chance could dominate effects of treatment on adverse events [15].

The main issues were the comparator treatments in trials and the dose of celecoxib. Pooling of data was therefore restricted to comparison between celecoxib and placebo, paracetamol, rofecoxib, and NSAIDs, because each comparator had a different mechanism of action from any other. In addition, analysis of celecoxib against all active comparators combined was carried out. For active comparisons, most of the information was likely to reside in those between celecoxib and NSAIDs, and we chose to perform two analyses: comparisons of all doses of celecoxib with all doses of NSAIDs, and between licensed daily doses of celecoxib and licensed doses of NSAIDs. NSAIDs were used at licensed doses, usually at maximum daily dose, and rofecoxib was used at 25 mg daily.

Information for osteoarthritis and rheumatoid arthritis was combined because the number of patients in trials with rheumatoid arthritis was small. Though there are differences between the conditions, notably age of onset, there are no clear reasons why treatment-emergent adverse events should differ between conditions. Analysis of celecoxib dose, and of duration of studies, was restricted to discontinuations due to lack of efficacy or to adverse events, where there were more than 20 events, and where the outcome had direct clinical relevance.

Analysis of data could potentially be performed in two ways. The simplest method would be to combine the absolute proportions of patients experiencing an adverse event, using the intention-to-treat population (randomised, at least one dose of drug) as the denominator. This method has a potential disadvantage of not taking into account different durations of studies, and possible different exposures between treatments because of different withdrawal rates. An alternative method would be to calculate adverse events as the rate of events occurring per year of exposure, theoretically taking both different durations and differential exposure into account.

This second method was impractical for several reasons. Trial reports generally did not have information to allow calculation of median duration of use. For instance, they reported neither average days of use nor individual days of use, so that an average could not be calculated. The reports generally had information on compliance, and generally there was no significant difference between celecoxib and its comparators. The two largest trials, with over half the patients, gave patient years of exposure in the trial reports, and these were identical for celecoxib and NSAID. In a separate analysis of cardiovascular events in celecoxib trials, which included 30,000 of the 40,000 patients in this review, there were negligible differences between treatment durations [16].

Outcomes were pooled in an intention-to-treat (number of patients randomised and receiving at least one dose of trial drug) analysis. Homogeneity tests and funnel plots, though commonly used in meta-analysis, were not used here because they have been found to be unreliable [17-19]. Instead clinical homogeneity was examined graphically [20]. Relative benefit (or risk) and number-needed-to-treat (or harm) were calculated with 95% confidence intervals. Relative risk was calculated using a fixed effects model [21], with no statistically significant difference between treatments assumed when the 95% confidence intervals included unity. We added 0.5 to celecoxib and comparator arms of trials in which at least one arm had no events. Number-needed-to-treat (or harm) was calculated by the method of Cook and Sackett [22], using the pooled number of observations.

Adverse outcomes were described in terms of harm or prevention of harm, as follows. When significantly fewer adverse events occurred with celecoxib than with a control substance (placebo or active), we used the term 'the number-needed-to-treat to prevent one event' (NNTp). When significantly more adverse events occurred with celecoxib than with an active comparator (paracetamol, rofecoxib, NSAID) we used the term 'number-needed-to-treat to harm one patient' (NNH).

## Results

### Trials

Clinical reports of 31 randomised trials – 21 in osteoarthritis, 4 in rheumatoid arthritis, and 6 in mixed osteoarthritis or rheumatoid arthritis – were provided for the analysis. Full company study reports for 23 trials contained 180,000 pages. These were comprehensive documents including detailed methods and results sections, tables, and figures. Appendices provided descriptions of the outcome measurement tools used, individual patient outcomes, compliance, case report forms, detailed statistical analyses, and protocol amendments. Full clinical trial reports were not available for eight trials, but extensive clinical trial summaries were provided. Information was extracted directly from the clinical trial reports or summaries.

All trials scored the maximum of five points for quality (Table 1), since they clearly described withdrawals in addition to the methods of randomisation and double blinding. All studies also scored the maximum of 16 points on the validity scale.

The 31 trials had 39,605 patients who were randomised and received at least one dose of study medication (intention-to-treat population). Of these, 25,903 had osteoarthritis, 3,232 had rheumatoid arthritis, and 10,470 were in trials including patients with both conditions. Sixteen of 21 trials in osteoarthritis (8,947 patients) lasted 2 to 6 weeks (13 lasted six weeks), and five (16,956 patients) lasted 12 weeks. One of the four trials (327 patients) in rheumatoid arthritis lasted 6 weeks, the other three (2,905 patients) lasted 12 or 24 weeks. Five trials in both osteoarthritis and rheumatoid arthritis (2,502 patients) lasted 12 weeks, and the other (7,968 patients) lasted 52 weeks (though the mean duration of exposure in all three treatment groups was about 7 months; 0.54 to 0.58 years). Most of the observations (77%) were therefore in trials of 12 weeks or longer.

Doses of celecoxib were 50 to 800 mg daily, mostly as twice-daily dosing. In trials of 2 to 6 weeks, 88% of the doses were 200 mg daily. In trials of 12 weeks' duration, 46% of doses were 200 mg and 46% were of 400 mg daily. In trials of 24 weeks or longer, 92% of doses were of 800 mg daily. Longer-lasting trials used higher doses of celecoxib. In comparisons with placebo, 88% of 6,857 patients taking celecoxib had doses in the licensed range of 200 to 400 mg daily. In comparisons with paracetamol and rofecoxib, the celecoxib dose was 200 mg daily. Analysis of licensed doses of celecoxib (200 to 400 mg daily) and NSAIDs not only avoided higher (800 mg) doses, but also the 52-week study that used 800 mg of celecoxib.

### Patients and adverse events

Details of the patients included in the trials are in Table 1. In most trials, the majority of patients were women whose average age was 60 years or above (range 17 to 96 years). The relevant medical history, notably about NSAID intolerance or gastrointestinal symptoms after use of NSAIDs and about use of prophylactic low-dose aspirin, was usually reported. Three trials (002, 149, 181) specifically recruited patients with stable, treated hypertension in addition to arthritis. Patients were predominantly Caucasian, but several studies specifically recruited only Asian participants, or those of mixed Asian, Afro-Caribbean, or Hispanic descent.

The adverse event outcomes measured in each trial are detailed in Additional file 3. All of the adverse events were those reported by trial investigators, and none was reported after independent, blinded adjudication.

Adverse events were measured by recording treatment-emergent events, clinical laboratory test results, or changes from baseline in vital signs found by physical examination. At each follow-up visit, patients were asked if they had experienced any symptoms not associated with their arthritis. Patients and study personnel were blinded to the identification of medication throughout the study, and if randomisation blind was broken, the patient was removed from the study.

### Discontinuation

**Figure 1:**
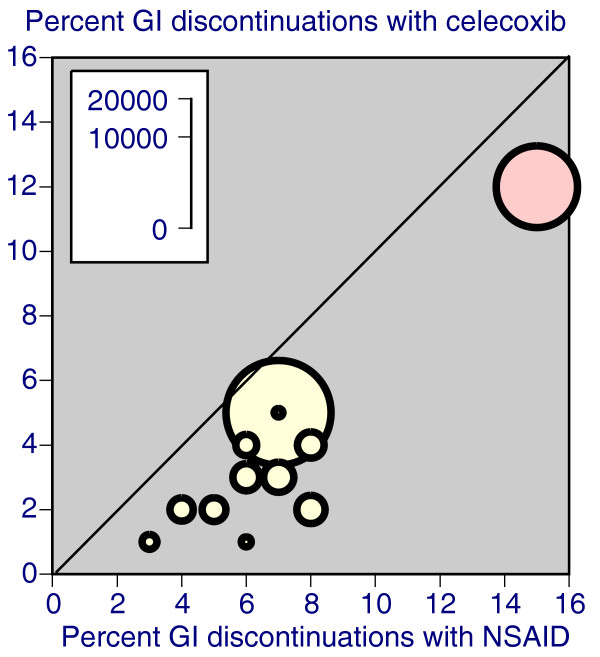
Scatter plot of trials comparing celecoxib with NSAID for discontinuations due to gastrointestinal adverse events. Celecoxib at any dose is represented. The red symbol represents the longest trial, at 52 weeks. GI, gastrointestinal; NSAID, nonsteroidal anti-inflammatory drug.

Details of discontinuations are shown in Table 2. All-cause and lack-of-efficacy discontinuations were less frequent with celecoxib than with placebo or paracetamol. Adverse-event and gastrointestinal-adverse-event discontinuation (Fig. 1) was less frequent with celecoxib than with NSAIDs (licensed dose or any dose) or any active comparator. All-cause discontinuations were also less frequent with any dose of celebcoxib compared with NSAID or any active comparator. Licensed doses of celebcoxib were not significantly different. Celecoxib did not differ from rofecoxib. The NNTp to prevent discontinuation due to lack of efficacy was 9 (8 to 11) compared with placebo, and 27 (14 to 390) compared with paracetamol. Licensed doses of celecoxib had an NNTp of 74 (47 to 180) for discontinuations due to an adverse event, and an NNTp of 58 (42 to 98) for discontinuations due to a gastrointestinal adverse event, compared with NSAIDs.

Proportions discontinuing because of lack of efficacy or adverse events varied according to drug, dose, and duration. Regarding duration, for instance, discontinuation because of gastrointestinal adverse events was higher for NSAIDs than celecoxib in the one 52-week trial and in trials of shorter duration (Fig. 1).

The details for all 39,605 patients in all trials are shown in Table 3. Discontinuation because of lack of efficacy was high with placebo, 18% over 2 to 6 weeks and 46% by 12 weeks. Effective treatment with licensed doses of celecoxib or NSAIDs reduced discontinuations due to lack of efficacy, with evidence of a dose-response for celecoxib over the range of 100 to 400 mg daily.

There was considerable variation between individual trials regarding discontinuations due to lack of efficacy at 12 weeks, for celecoxib and naproxen. The variability seemed unrelated to condition, and no sensible reason presented itself.

Discontinuations due to adverse events were low with placebo (6% at 12 weeks), little different with celecoxib, and somewhat higher with NSAIDs (Tables 2 and 3). In trials of 24 weeks or longer, discontinuations due to adverse events with 800 mg celecoxib, 100/150 mg diclofenac, and 2,400 mg ibuprofen were between 22% and 26%.

### Any adverse event

The proportion of patients reporting any adverse event was of the order of 50% (Table 4). Patients taking celecoxib reported adverse events more frequently than those taking placebo (NNH 15; 11 to 21), and less frequently than with NSAIDs (NNTp 18; 14 to 23 for licensed doses) or any active comparator. There was no difference between celecoxib and either paracetamol or rofecoxib.

### Treatment-related adverse events

About one-third of all reported adverse events were considered to be treatment related (Table 4). There was no difference between celecoxib and paracetamol or rofecoxib. More patients taking celecoxib than placebo had a treatment-related adverse event (NNH 71; 39 to 450). Fewer patients experienced a treatment-related adverse event with celecoxib than with NSAID (NNTp 24; 19 to 31 for licensed doses) or any active comparator.

### Serious adverse events

**Figure 2:**
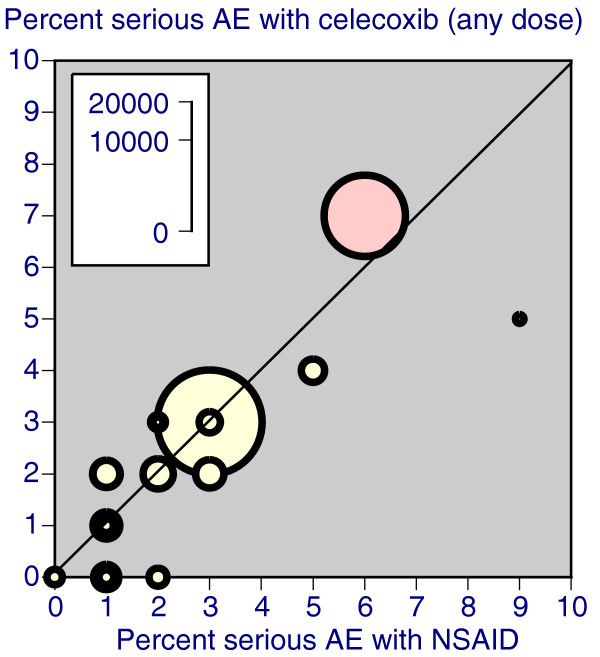
Scatter plot of trials comparing any dose of celecoxib with NSAID for serious adverse events. The red symbol represents the longest trial, at 52 weeks. AE, adverse events; NSAID, nonsteroidal anti-inflammatory drug.

The proportion of patients with a serious adverse event was low, averaging 1 to 3% (Table 4). Fewer patients taking celecoxib than placebo had serious adverse events (NNTp 280; 120 to 790). There was no difference in serious adverse event rates for celecoxib compared with paracetamol, rofecoxib, NSAID (Fig. 2), or any active comparator (Table 4). Serious adverse events occurred more often, at 6%, in the single 52-week trial than in trials of shorter duration (Fig. 2), but not more often than with NSAID.

### Any gastrointestinal adverse event

The proportion of patients reporting any gastrointestinal adverse event was of the order of 25% (Table 4). More patients taking celecoxib than placebo reported a gastrointestinal adverse event (NNH 14; 12 to 19). There was no difference between celecoxib and either paracetamol or rofecoxib. Celecoxib had fewer patients reporting any gastrointestinal adverse event than either NSAID (NNTp 12; 10 to 13 for licensed doses) or any active comparator.

### Gastrointestinal tolerability

Gastrointestinal tolerability (the proportion of patients having moderate or severe nausea, dyspepsia, or abdominal pain) was about 5% with celecoxib (Table 5). There was no difference between celecoxib and placebo, paracetamol, or rofecoxib. Celecoxib had less gastrointestinal intolerance than NSAIDs (NNTp 28; 24 to 36 for licensed doses of celecoxib) or any active comparator.

### Nausea

The proportion of patients reporting nausea was about 3% with celecoxib (Table 5a). Nausea was significantly lower with celecoxib than placebo (NNTp 155; 71 to 840), and for celecoxib at any dose compared with NSAID or any active comparator. There was no difference between celecoxib and paracetamol, or rofecoxib, or between licensed doses of celecoxib and NSAIDs.

### Vomiting

The proportion of patients experiencing vomiting was about 1% with celecoxib (Table 5a). There was no difference between celecoxib and placebo, paracetamol, or rofecoxib. Celecoxib at both licensed dose and any dose had fewer patients with vomiting than NSAID (NNTp 173; 115 to 350 for licensed doses) or any active comparator.

### Abdominal pain

The proportion of patients reporting abdominal pain was about 5% with celecoxib (Table 5b). There was no difference between celecoxib and placebo, or paracetamol. Celecoxib (any dose) produced less abdominal pain than rofecoxib 25 mg (NNTp 67; 35 to 920). Celecoxib at both licensed dose and any dose had fewer patients reporting abdominal pain than NSAID (NNTp 41; 32 to 57 for licensed doses) or any active comparator.

### Dyspepsia

The proportion of patients reporting dyspepsia was about 7% with celecoxib (Table 5b). Celecoxib (any dose) produced more dyspepsia than placebo (NNH 46; 32 to 84). There was no difference between celecoxib and paracetamol, or rofecoxib. Celecoxib at both licensed and any dose had fewer patients reporting dyspepsia than NSAID (NNTp 61; 43 to 100 for licensed doses) or any active comparator.

### Diarrhoea

The proportion of patients experiencing diarrhoea was about 6% with celecoxib (Table 5b). Celecoxib (any dose) produced more diarrhoea than placebo (NNH 53; 37 to 97). Celecoxib (any dose) produced less diarrhoea than paracetamol 4,000 mg (NNTp 41; 22 to 450). There was no difference between celecoxib and rofecoxib, or between celecoxib (at the licensed dose or any dose) and NSAID, or any active comparator.

### Clinical ulcers and bleeds

Clinical ulcers and bleeds in the company clinical trial reports were as reported by investigators, and were not subjected to independent, blinded adjudication in trials where this was not a primary outcome. The proportion of patients having a clinical ulcer or bleed was under 0.5% with celecoxib (Table 5b). No analysis was possible for clinical ulcers and bleeds for the comparisons between celecoxib and placebo, paracetamol, and rofecoxib, as there were only three events, no events, and one event, respectively. Celecoxib at both the licensed dose and any dose had fewer patients with clinical ulcers and bleeds than NSAID (NNTp 250; 170 to 450 for licensed doses) or any active comparator.

### Myocardial infarction

Myocardial infarction in the company clinical trial reports was as reported by investigators, and was not subjected to independent, blinded adjudication. The numbers of reported myocardial infarctions in each arm of each trial are given in Additional file 3.

The proportion of patients having a myocardial infarction was under 0.3% with celecoxib (Table 6). No analysis was possible for myocardial infarction for the comparisons between celecoxib and placebo, paracetamol, and rofecoxib, as there were only 10 events, no events, and 1 event, respectively. Proportions for celecoxib at both the licensed dose and any dose were not significantly different from NSAID, any active comparator, any active comparator excluding rofecoxib, or any comparator, including both rofecoxib and placebo.

The numbers of events were small, with fewer than 60 cases of myocardial infarction in the largest comparison. Most trials had either no cases of myocardial infarction, or a single case in one of the treatment arms. No analysis demonstrated a statistical difference between celecoxib and any comparator (Table 6). For the comparison of all celecoxib doses with all comparators except rofecoxib, the number of events was 39/20,933 (0.19%) for celecoxib and 18/15,383 (0.12%) for comparators. For the comparison of licensed doses of celecoxib with NSAID, the number of events was 20/13,509 (0.15%) for celecoxib 200 to 400 mg daily and 3/8,309 (0.04%) for NSAID.

Forty-four cases of myocardial infarction occurred in the two largest trials (096 and 102), with 21,162 patients. Their planned duration was 12 and 52 weeks, and they had a combined actual duration of about 4.5 months. Here 29/12,787 (0.23%) of patients taking celecoxib (200 to 800 mg) suffered a myocardial infarction, compared with 15/8,375 (0.18%) on NSAID. The relative risk was 1.7 (0.88 to 3.2). Calculating the NNH gave a figure of 2,100 with a 95% confidence interval of 588 patients harmed to 1,337 patients where harm was prevented.

### Cardiac failure

The proportion of patients with cardiac failure was under 0.2% with celecoxib (Table 6). No analysis was possible for the comparisons between celecoxib and placebo, paracetamol, and rofecoxib, as there were only 5 events, no events, and 10 events, respectively. Proportions for celecoxib at both the licensed dose and any dose were not significantly different from NSAID or any active comparator.

### Raised creatinine

For the incidence of creatinine raised to 1.3 times the upper limit of normal or more, data were available only for the comparisons between celecoxib and placebo, celecoxib at licensed doses and NSAID, and celecoxib compared with any active comparator. There were no significant differences (Table 6). The proportion of any patient having raised creatinine was up to 1% with celecoxib.

### Hypertension and aggravated hypertension

This outcome combined a new diagnosis of hypertension with aggravated hypertension in patients with an existing diagnosis of hypertension, but in whom changed or additional treatment was needed for control of hypertension. The proportion of any patient having hypertension or aggravated hypertension was 1 to 2% with celecoxib (Table 6). There was no significant difference between celecoxib and any comparator, placebo, rofecoxib, or NSAIDs. For paracetamol there were only four events.

### Oedema at any site

Oedema was reported in various ways in the trials, occasionally just as oedema, sometimes broken down by body site. The proportion of patients with oedema was usually about 3% (Table 6), but it was much higher at 23 to 38% in two trials (149, 181) in patients with osteoarthritis and treated hypertension, with oedema as a predefined end point. Proportions were 5 to 10% in another trial (002) in patients with osteoarthritis, diabetes, and hypertension, also with oedema as a predefined end point.

**Figure 3:**
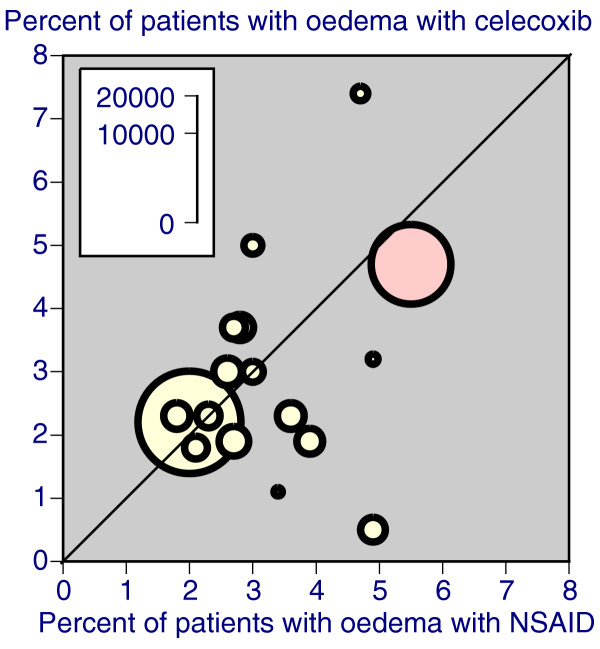
Scatter plot of trials comparing any dose of celecoxib with NSAID for oedema. The red symbol represents the longest trial, at 52 weeks. NSAID, nonsteroidal anti-inflammatory drug.

Celecoxib was associated with significantly more oedema than placebo (NNH 79; 54 to 145). Celecoxib was no different from paracetamol. Celecoxib (200 mg daily) had significantly less oedema than rofecoxib (25 mg daily), with an NNTp of 14 (10 to 25). Celecoxib at licensed doses or at any dose was no different from NSAID for oedema (Fig. 3), but was significantly better than any active comparator (NNTp 62; 48 to 87).

### Haemoglobin fall of 20 g/L or more

This parameter was not reported in studies comparing celecoxib with paracetamol or rofecoxib. The incidence of a haemoglobin fall of 20 g/L or more was about 1% with celecoxib (Table 7). There was no difference between celecoxib and placebo. Celecoxib at both the licensed dose and any dose had a lower incidence than NSAID (NNTp 92; 66 to 150 for licensed doses) or any active comparator.

### Haematocrit fall of 5% or more

This parameter was not reported in studies comparing celecoxib with paracetamol. The incidence of a haematocrit fall of 5% or more was about 10% with celecoxib (Table 7). There was no difference between celecoxib and placebo or rofecoxib. Celecoxib at both the licensed dose and any dose had a lower incidence than NSAID (NNTp 18; 14 to 25 for licensed doses) or any active comparator.

### Endoscopically detected ulcers

**Figure 4:**
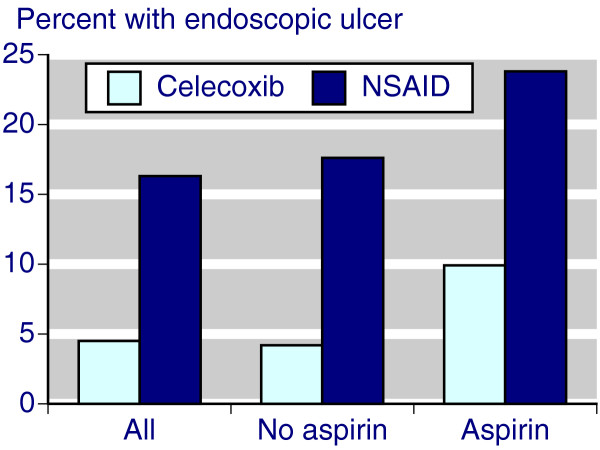
Endoscopically identified ulcers in patients taking celecoxib and NSAID, with and without prophylactic low-dose aspirin. NSAID, nonsteroidal anti-inflammatory drug.

Seven trials were designed to ascertain the presence of endoscopically detectable ulcers of 3 mm or more; in these, celecoxib was compared with placebo and/or NSAID (Additional file 4). Six reported at 12 weeks, and one at 24 weeks. Five trials also reported results according to the use of low-dose aspirin of 325 mg or less daily. These results are shown in Table 8 and Fig. 4, analysed across all patients and according to aspirin use. In no comparison was there any significant difference between celecoxib and placebo. For both celecoxib and NSAID, there was the same 6% absolute increase in endoscopically detected ulcers with aspirin use. Celecoxib, at both the licensed dose and any dose, always produced more endoscopically detected ulcers than NSAID. The NNTp was the same at 7 to 8 both with and without concomitant aspirin use.

### Deaths

There were 28 deaths during the trials or within 28 days of stopping medication, of which 21 were cardio/cerebrovascular, 1 was of unknown cause, and 6 were due to other causes. We included the unknown with the cardiovascular deaths for analysis. The incidence with celecoxib was 0.01% (1/6,844) compared with 0.03% (1/3,060) with placebo, and 0.01% (1/13,904) with licensed doses of celecoxib compared with 0.07% (6/8,704) with NSAIDs. When all doses of celecoxib were analysed, the incidence was 0.03% (6/18,325), compared with 0.11% (14/12,685) with NSAIDs and 0.10% with all active comparators.

## Discussion

There have been a number of systematic reviews of published papers of coxibs in arthritis, and several have examined specific adverse events. Serious upper gastrointestinal events in phase II and III studies were reported for rofecoxib [23] and celecoxib [24]. Others have looked at renal [25] or cardiac adverse events [26]. Cochrane reviews of cyclooxygenase inhibitors in rheumatoid arthritis have limited information to date on efficacy and safety of rofecoxib [27], and only five trials with 5,400 patients taking celecoxib [28]. Two previous systematic reviews of coxibs used company clinical trial reports. Deeks and co-workers [29] examined 15,000 patients in nine of the earlier trials of celecoxib, and Edwards and co-workers [30] examined some 5,700 patients in nine trials of valdecoxib.

Reviews looking at adverse events generally [29,30] have analysed adverse events by combining the absolute proportions of patients experiencing an adverse event, using the intention-to-treat population (randomised, at least one dose of drug) as the denominator. Those examining particular, rare adverse events (gastrointestinal bleeding, cardiovascular events) have tended to use exposure correction, together with independent blinded adjudication of the event [16,25,26].

This systematic review greatly increases the quantity and quality of information available on adverse events with celecoxib in arthritis. We had data from 31 trials, with almost 40,000 patients. The individual trials all scored the maximum on two systems for scoring reporting quality and validity in pain trials. Use of similar methods for collecting and reporting adverse events ensured data of uniform nature and quality.

The average age in the trials was about 60 years (Table 1), but there was a wide range (17 to 96 years). Several studies recruited special groups, for instance, patients with diabetes or hypertension, or patients who were solely Asian, or of mixed Asian, Afro-Caribbean, or Hispanic descent. Most trials documented relevant medical history, such as previous NSAID use or intolerance, or use of prophylactic low-dose aspirin. While non-Caucasians were under-represented, and many patients with significant comorbidities were excluded from the trials, this population is probably as representative as possible in clinical trials.

This gives credibility to the review in terms of size, quality, and validity, allowing us to make sense of all but the most rare adverse event. At the same time, there are limitations.

Multiple comparisons could be made, including condition treated, duration of study, comparator drug, and dose. Ideally all these would be tested by sensitivity analysis. We limited our analyses to comparator and dose to avoid excessive subdivision and proliferation of statistical testing, which can lead to spurious statistical significance [31]. Analysis by condition or duration was avoided because few patients (8%) were in trials with rheumatoid arthritis only, and few observations (23%) were made in trials lasting less than 12 weeks. Instead we concentrated on analysis by comparator, where there was the possibility of major differences based on large amounts of high-quality experimental evidence, and on dose. Most doses were in the licensed range, but for completeness we chose to perform analyses of celecoxib versus NSAID by all doses, and those within the licensed range.

Generally, trial reports indicate that World Health Organization Adverse Reaction Terminology criteria were used to define adverse events, but these are not immediately accessible. For any particular treatment-emergent adverse event, we have had to assume that the same criteria were used consistently both within and between trials. Although adequate clinical trial monitoring makes this highly probable, we have no positive evidence that this was the case. Definitions are problematical for reporting adverse events [32,33].

**Figure 5:**
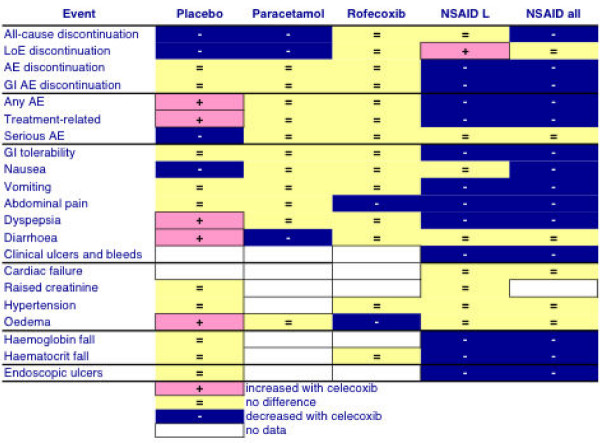
Summary of main comparisons of treatment of arthritis with celecoxib or other drugs and placebo. AE, adverse event; GI, gastrointestinal; L, licensed doses; LoE, lack of efficacy; NSAID, nonsteroidal anti-inflammatory drug.

The statistical direction of the results for each adverse event outcome and each comparison is shown in Fig. 5.

In comparison with placebo (10,000 patients), celecoxib had fewer all-cause and lack-of-efficacy discontinuations, but more adverse events. Lower discontinuations result from greater efficacy, but an active drug at an effective therapeutic dose is likely to produce some adverse events. Importantly, there was no difference in gastrointestinal tolerability or endoscopically detected ulceration.

Only two trials (1,056 patients) compared celecoxib (200 mg/day) with paracetamol 4,000 mg/day. There were fewer all-cause and lack-of-efficacy discontinuations with celecoxib, and almost identical adverse event profiles, indicating better efficacy with no excess harm. It is worth noting that recent large randomised comparisons of paracetamol with placebo over 12 weeks have failed to show any better efficacy for paracetamol than placebo [34].

Five trials (2,671 patients) compared celecoxib (200 mg/day) with rofecoxib (25 mg/day). Celecoxib had less abdominal pain and oedema. Rofecoxib is another cyclooxygenase-2 selective inhibitor, and similarity between their adverse event profiles is to be expected.

In the comparisons with NSAIDs, the better adverse event profile of celecoxib was marked, both at licensed doses (23,000 patients) and any dose (31,000 patients). There were more discontinuations for lack of efficacy with celecoxib at licensed doses than with NSAIDs, balanced by fewer adverse-event discontinuations or gastrointestinal-adverse-event discontinuations. There were fewer adverse events overall, treatment-related adverse events, combined and individual gastrointestinal adverse events, with the exception of diarrhoea, but including gastrointestinal tolerability, and endoscopically detected ulcers. There were also possible benefits relating to loss of blood in the lower gastrointestinal tract, with fewer patients having falls in haemoglobin or haematocrit. These results again are expected, and are similar to results for celecoxib, valdecoxib, and rofecoxib in recent analyses and a trial [35-37].

Cyclooxygenase-2 selective inhibitors are known to produce fewer upper gastrointestinal ulcers and bleeds [38-42], and less gastrointestinal upset [43], than NSAIDs. The results here confirm this for celecoxib. For gastrointestinal tolerability (moderate or severe nausea, dyspepsia, or abdominal pain), one patient fewer would suffer for every 28 treated with celecoxib than with NSAID. One in 17 would not have a haematocrit fall of 5% or more.

The lack of difference between celecoxib and NSAIDs with regard to cardio-renal adverse events is not unexpected. There are no known benefits for cyclooxygenase-2 selective inhibitors over nonspecific inhibitors relating to cardiac or renal function, and the known associations between NSAID use and renal failure [8] and heart failure [10,11] are likely to apply to cyclooxygenase-2 selective inhibitors.

**Figure 6:**
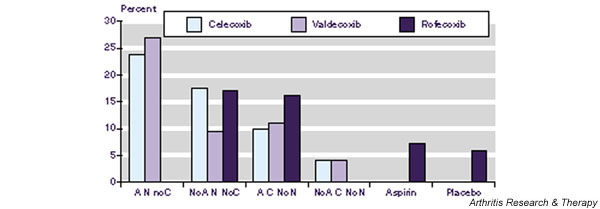
Endoscopically identified ulcers after treatment of arthritis with placebo, low-dose aspirin, NSAID, coxibs, and combinations. A, aspirin; N, NSAID; NSAID, nonsteroidal anti-inflammatory drug; C, coxib.

Endoscopically detected ulcers were affected both by whether celecoxib or NSAID was used, and by whether or not prophylactic low-dose aspirin was used (Table 8). The number-needed-to-treat to prevent one endoscopically detected ulcer was about 7, with or without aspirin. The protective effect of celecoxib was the same whether aspirin was present or not, and use of aspirin increased endoscopically detected ulcers by the same absolute incidence of 6%. This was nearly identical to results found in a systematic review of studies of valdecoxib in arthritis [30], but different comparisons make it difficult to know whether rofecoxib is different [37] (Fig. 6). The much lower incidence of endoscopically detected ulceration with celecoxib compared with NSAID reflected a similar result for rofecoxib [44,45], though the rofecoxib studies had no patients using aspirin. What is clear is that celecoxib plus low-dose aspirin produces no more endoscopically detected ulcers than NSAID without aspirin, and fewer than NSAID plus aspirin.

On maximum-dose NSAID, or celecoxib, or paracetamol, up to 30% of patients withdrew from treatment. The main reasons were lack of efficacy or adverse events. Withdrawals increased with duration of study, as would be expected (Table 2). They were also influenced by drug and dose (Table 2), though small numbers of events hindered comparisons. The tendency for fewer withdrawals with celecoxib than NSAID mirrors what has been found in clinical practice [46], though not in clinical trials of valdecoxib [30], based on many fewer patients than in this review. Overall medical costs of cyclooxygenase-2 selective inhibitors are not different from those of NSAIDs [46,47], because higher acquisition costs of cyclooxygenase-2 selective inhibitors appear to be balanced by higher costs of treating or preventing adverse events with NSAIDs.

Even with as large a data set as here, some rare but serious adverse events occur in so few people that it is difficult to determine whether apparent differences (significant or nonsignificant) between treatments are real or meaningful. Examples are cardiac failure, myocardial infarction, and death, with total maximum numbers of 55, 59, and 28 respectively. The incidence of these events was of the order of 0.3 per 1,000 patients to 2 per 1,000 patients. Cardiac failure and death with celecoxib were lower than with NSAIDs (but not significantly), while myocardial infarction rates were higher (but not significantly). Incidence may be additionally affected by exposure bias, different exposure with different treatments (Table 2). Analysis correcting for exposure bias may then be more appropriate [16], even though there appears to be little exposure bias between celecoxib and NSAIDs in arthritis trials.

Where adverse events are rare, even large numbers of patients in randomised, controlled trials will accumulate few events. If such trials are of relatively short duration, then there is even less opportunity to accumulate these rare events. In the 31 trials in this review, the longest duration of exposure was an average of about 7 months, and most were less than 3 months. The consequence is a residual uncertainty, as here for attributing higher risk of myocardial infarction with celecoxib than with other non-coxib comparators. Limitations in number of events and duration of constituent trials means that any possible relationship between celecoxib and myocardial infarction cannot be completely dispelled by these data alone, despite lack of a statistically significant difference.

## Conclusion

This review of a large number of randomised trials and patients provides more accurate estimates of frequency and more confidence in the adverse event pattern. These are likely to be the minimum expected in clinical practice, where the population may be sicker, or take more medications, than in clinical trials.

Using company clinical trial reports removes some of the problems of selective reporting in published papers due to strict word limitations. Here the company clinical trial reports and extensive trial summaries provided about five pages of information per patient. While efficacy in published studies is poorly presented [48], it is available in clinical reports [49]. Information about adverse events is even more poorly presented in published papers [50], but it is clearly presented in company clinical trial reports. Company clinical trial reports represent an ideal source of information for systematic review and meta-analysis.

## Abbreviations

NNH = number-needed-to-harm; NNT = number-needed-to-treat; NNTp = number-needed-to-treat to prevent one event; NSAID = nonsteroidal anti-inflammatory drug.

## Competing interests

RAM and HJM have received lecture fees from pharmaceutical companies. The authors have received research support from charities and government sources at various times. GM is an employee of Pfizer. This work was supported by an unrestricted educational grant from Pfizer Ltd. The terms of the financial support from Pfizer included freedom for authors to reach their own conclusions, and an absolute right to publish the results of their research, irrespective of any conclusions reached. Pfizer did have the right to view the final manuscript before publication, and did so. No author other than GM has any direct stock holding in any pharmaceutical company.

## Authors' contributions

RAM was involved with planning the study, data extraction, analysis, and preparing the manuscript; SD with data extraction, analysis, and writing; GTM with planning, data extraction, analysis, and writing the manuscript; HJM with planning, analysis, and writing. All authors read and approved the final manuscript.

## Supplementary Material

Additional File 1 A Word file containing a table giving trial inclusion and exclusion criteria for arthritis. Click here for file

Additional File 2 A Word file containing a table giving definitions of adverse events. Click here for file

Additional File 3 An Adobe Acrobat file containing a table showing details of treatment, discontinuations, and adverse events. Click here for file

Additional File 4 An Adobe Acrobat file containing a table showing detailed endoscopic outcomes. Click here for file

## Figures and Tables

**Table 1:** Included studies of tolerability, adverse events, and endoscopically detected ulceration associated with celecoxib in arthritis

			Drug, dose, number randomised						
Study	Details of participants	Relevant medical history	Celecoxib	Placebo	Other	Duration (weeks)	Efficacy outcomes	Safety outcomes	Total in trial (ITT)

***Osteoarthritis***									

C-002	OA Hip/Knee (ACR) requiring daily NSAID therapy, FCC 1–3 Stable hypertension, type 2 diabetes Age 62 (range 40–89) years 61% female ≥ 75% Caucasian	Data not provided	1 × 200 mg/day, *n *= 36	No placebo	Rofecoxib 1 × 25 mg/day, *n *= 132 Naproxen 2 × 500 mg/day, *n *= 128	12	WOMAC Patient's assessment of arthritis pain VAS Patient's global assessment of arthritis Patient's satisfaction Withdrawal due to lack of efficacy	Withdrawals Adverse events Serious adverse events Laboratory tests	396
C-003	OA Knee (ACR) with flare, requiring daily NSAID/analgesic, FCC 1–3, baseline pain 40 on 100 mm VAS. Age 63 (range 39–90) years Duration of disease 8 (range 0.2–51) years 67% female ≥ 85% Caucasian	Cardioprotective ASA 20% NSAID intolerance 4% GI ulcer 6% GI bleed 1% Renal insufficiency 1%	1 × 200 mg/day, *n *= 189	*n *= 96	Rofecoxib 1 × 25 mg/day, *n *= 190	6	Patient's assessment of arthritis pain WOMAC (total) Patient's global assessment of arthritis pain VAS OASI Physician's global assessment of arthritis Patient's assessment of satisfaction	Withdrawals Adverse events Serious adverse events Laboratory tests	475
C-010	OA Hip/Knee (K-L 2–4), requiring chronic NSAID/analgesic, initial pain 40–90 on 100 mm VAS Age 63 (range 38–91) years Duration of disease 9 (0.1–54) years 62% female	Cardioprotective ASA 20% GI-related NSAID intolerance 1% Gastroduodenal ulcer 8% GI bleed 0.6% Some type of GI history (unspecified) 48%	1 × 200 mg/day *n *= 181	n = 172	Paracetamol 4 × 1,000 mg/day, *n *= 171	6	WOMAC index MDHAQ Patient's global rating of helpfulness Physician's global assessment of status SF-36 General clinical safety	Withdrawals Adverse events Serious adverse events Laboratory tests	524
C-013	OA Knee (ACR) with flare, FCC 1–3 Mean age 62 (range 29–92) years Duration of disease 10 (0.2–50) years 69% female 90% Caucasian	Cardioprotective ASA permitted NSAID intolerance 13% Gastroduodenal ulcer 16% GI bleed 3% CVD 52%	2 × 40 mg/day, *n *= 73 2 × 100 mg/day, *n *= 75 2 × 200 mg/day, *n *= 73	*n *= 70	No active comparator	2	Physican's global assessment Patient's global assessment Patient's arthritis pain SF-36	Withdrawals Adverse events Serious adverse events Laboratory tests	291
C-020	OA knee/hip (ACR) with flare, FCC 1–3 Age 62 (range 21–89) years Duration of disease 9 (0.1–52) years 66% female 79% Caucasian	Cardioprotective ASA permitted NSAID intolerance 7% Gastroduodenal ulcer 9% GI bleed 2% CVD 53%	2 × 50 mg/day, *n *= 218 2 × 100 mg/day, *n *= 217 2 × 200 mg/day, *n *= 222	*n *= 219	Naproxen 2 × 500 mg/day, *n *= 216	12	Patient's global assessment Physician's global assessment WOMAC Patient's assessment of pain	Withdrawals Adverse events Serious adverse events Laboratory tests	1,092
C-021	OA Knee/Hip (ACR) with flare, FCC 1–3 No ulcer at baseline endoscopy Age 61 (range 22–89) years Duration of disease 9 (range 0.1–52) years 54% female 83% Caucasian	Cardioprotective ASA permitted. NSAID intolerance 10% Gastroduodenal ulcer 17% GI bleed 2% CVD 60%	2 × 50 mg/day, *n *= 258 2 × 100 mg/day, *n *= 239 2 × 200 mg/day, *n *= 237	*n *= 247	Naproxen 2 × 500 mg/day, *n *= 233	12	Patient's global assessment Patient's assessment of pain Physician's global assessment WOMAC	Withdrawals Adverse events Serious adverse events Laboratory tests Endoscopic ulcers	1,214
C-042	Symptomatic OA Hip/Knee (ACR) ≥ 6 months, requiring NSAID, FCC 1–3 Age 63 (range 34–91) years Duration of disease 7 (0.5–48) years 72% female 94% Caucasian	NSAID intolerance 2% Gastroduodenal ulcer 3% GI bleed 0.5% CVD 45%	2 × 100 mg/day, *n *= 346	No placebo	Diclofenac 2 × 50 mg/day, *n *= 341	6	Patient's global assessment Patient's assessment of pain Physician's global assessment SF-36	Withdrawals Adverse events Serious adverse events Laboratory tests	667
C-047	OA Knee (ACR) with flare, FCC 1–3 Age 63 (29–91) years Duration of disease 9 (0.5–60) years 72% female 84% Caucasian	Cardioprotective ASA permitted NSAID intolerance 10% Gastroduodenal ulcer 10% GI bleed 4% CVD 62%	2 × 25 mg/day, *n *= 100 2 × 100 mg/day, *n *= 101 2 × 400 mg/day, *n *= 99	*n *= 101	No active comparator	4	Patient's global assessment Patient's assessment of pain Physician's global assessment SF-36 WOMAC	Withdrawals Adverse events Serious adverse events Laboratory tests	401
C-054	OA Hip (ACR) with flare, FCC 1–3 Age 62 (28–93) years Duration of disease 7 (0.1–64) years 66% female 92% Caucasian	Cardioprotective ASA permitted NSAID intolerance 13% Gastroduodenal ulcer 12% GI bleed 2% CVD 60%	2 × 50 mg/day, *n *= 216 2 × 100 mg/day, *n *= 207 2 × 200 mg/day, *n *= 213	*n *= 217	Naproxen 2 × 500 mg/day, *n *= 207	12	Patient's global assessment Patient's assessment of pain Physician's global assessment SF-36 WOMAC	Withdrawals Adverse events Serious adverse events Laboratory tests	1,060
C-060	OA Knee (ACR) with flare, FCC 1–3 Age 63 (29–88) years Duration of disease 9 (0.1–59) years 66% female 88% Caucasian	Cardioprotective ASA permitted NSAID intolerance 4% Gastroduodenal ulcer 6% GI bleed 2% CVD 58%	2 × 100 mg/day, *n *= 231 1 × 200 mg/day, *n *= 222	*n *= 231	No active comparator	6	Patient's global assessment Patient's assessment of pain Physician's global assessment SF-36 WOMAC	Withdrawals Adverse events Serious adverse events Laboratory tests	684
C-087	OA Knee (ACR) with flare, FCC 1–3 Age 61 (18–89) years Duration of disease 9 (0.1–60) years 70% female 86% Caucasian	Cardioprotective ASA permitted NSAID intolerance 7% Gastroduodenal ulcer 16% GI bleed 2% CVD 64%	2 × 100 mg/day, *n *= 241 1 × 200 mg/day, *n *= 231	*n *= 243	No active comparator	6	Patient's global assessment Patient's assessment of pain Physician's global assessmen WOMAC	Withdrawals Adverse events Serious adverse events Laboratory tests	715
C-096	OA Knee/Hip/Hand ≥ 6 months (ACR) requiring daily analgesic/ NSAID, FCC 1–3 Age 62 (range 21–96) years 76% female Duration of disease 7 (0.3–59) years	Cardioprotective ASA use 7% CVD 41% Renal insufficiency 0.2% Respiratory disease 5% Diabetes 8%	2 × 100 mg/day, *n *= 4,393 2 × 200 mg/day, *n *= 4,407	No placebo	Naproxen 2 × 500 mg/day, *n *= 905 Diclofenac 2 × 50 mg/day, *n *= 3,489	12	Patient's global rating of arthritis Patient's assessment of pain (VAS) WOMAC Physician's global assessment of arthritis	Withdrawals Adverse events Serious adverse events Laboratory tests	13,194
C-118	OA Knee (ACR) with flare, FCC 1–3 Age 61 (29–88) years Duration of disease 8 (0.1–62) years 65% female 82% Caucasian	Cardioprotective ASA permitted NSAID intolerance 3% Gastroduodenal ulcer 8% GI bleed 1% CVD 66%	2 × 100 mg/day, *n *= 199	*n *= 200	Diclofenac 3 × 50 mg/day, *n *= 199	6	Patient's global assessment Patient's assessment of pain Physician's global assessment WOMAC	Withdrawals Adverse events Serious adverse events Laboratory tests	598
C-149	OA Hip/Knee/Hand (ACR) requiring NSAID, FCC 1–3 Stable treated hypertension Age 74 (range 64–95) years Duration of disease range 0.3–61 years 67% female Majority Caucasian	Cardioprotective ASA 38% NSAID intolerance 3% Gastroduodenal ulcer 10% GI bleed 3% Oedema 26% CHF 5%	1 × 200 mg/day, *n *= 411	No placebo	Rofecoxib 25 mg/day, *n *= 399	6	Oedema Aggravated hypertension Renal events	Withdrawals Adverse events Serious adverse events Laboratory tests	810
C-152	OA Knee (ACR) with flare, FCC 1–3, baseline pain 35 on 100 mm VAS Age 62 (range 40–88) years Duration of disease 11 (range 0.5–s47) years 71% female 80% Caucasian	Cardioprotective ASA permitted NSAID intolerance 4% Gastroduodenal ulcer 9% GI bleed 0.5%	1 × 200 mg/day, *n *= 63	*n *= 60	Rofecoxib 1 × 25 mg/day, *n *= 59	6	Patient's assessment of arthritis pain OA VAS scale Patient's global assessment of arthritis WOMAC	Withdrawals Adverse events Serious adverse events Laboratory tests	182
C-181	OA Hip/Knee/Hand (ACR) requiring daily NSAID, FCC 1–3 Stable treated hypertension Age 73 (range 65–96) years Duration of disease 12 (0–63) years 62% female 88% Caucasian	Cardioprotective ASA permitted NSAID intolerance 2% Gastroduodenal ulcer 8% GI bleed 2% Oedema 27% CHF 3%	1 × 200 mg/day, *n *= 549	No placebo	Rofecoxib 1 × 25 mg/day, *n *= 543	6	Blood pressure Oedema Weight Anti-hypertensive medication	Withdrawals Adverse events Serious adverse events Laboratory tests	1,092
C-209	OA Knee with flare (ACR), requiring chronic NSAID, FCC 1–3, initial pain 40–90 on 100 mm VAS Age 58 (range 45–83) years Duration of disease 5 (range 0.1–36) years 80% female Afro-American population	Data not provided	1 × 200 mg/day, *n *= 125	*n *= 66	Naproxen 2 × 500 mg/day, *n *= 125	6	Patient's assessment of arthritis pain Patient's global assessment Physician's global assessment WOMAC	Withdrawals Adverse events Serious adverse events Laboratory tests	316
C-210	OA Knee (ACR) with flare, FCC 1–3, requiring daily therapy, baseline pain 40–90 on 100 mm VAS Age 65 (range 42–90) years 68% female Duration of disease 5 (0.3–38) years Asian American population 100% Asian descent	Data not provided	1 × 200 mg/day, *n *= 145	*n *= 76	Naproxen 2 × 500 mg/day, *n *= 141	6	Patient's assessment of arthritis pain Patient's global assessment Physician's global assessment Pain Satisfaction WOMAC	Withdrawals Adverse events Serious adverse events Laboratory tests	362
C-211	OA Knee (ACR) with flare, requiring daily NSAID, FCC 1–3, baseline pain 40–90 on 100 mm VAS Age 60 (range 40–88) years Duration of disease 6 (range 0.1–36 yrs) years 67% female Hispanic population	Data not provided	1 × 200 mg/day, *n *= 125	*n *= 61	Naproxen 2 × 500 mg/day, *n *= 129	6	Patient's assessment of arthritis pain Patient's global assessment Physician's global assessment WOMAC Patient's satisfaction	Withdrawals Adverse events Serious adverse events Laboratory tests	315
C-216	OA Knee, symptomatic, requiring NSAID, initial pain 40 on 100 mm VAS Age 63 (range 20–92) years Duration of disease 4 (range 0.1–37) years 66% female Asian population	Cardioprotective ASA 3% NSAID intolerance 0.1% GI bleed 0.2% Gastroduodenal ulcer 6% CVD 30%	2 × 100 mg/day, *n *= 382	*n *= 192	Loxoprofen 3 × 60 mg/day, *n *= 385	4	Final global improvement rating Patient's assessment of arthritis pain Physician's and patient's global assessment of arthritis WOMAC	Withdrawals Adverse events Serious adverse events Laboratory tests Global safety rating	959
C-249	OA Hip/Knee (K-L confirmed), baseline pain 40–90 on 100 mm VAS Age 63 (range 45–89) years Duration of disease 9 (range 0.1–50) years 66% female ≥ 80% Caucasian	Cardioprotective ASA 21% GI-related NSAID intolerance 2% Gastroduodenal ulcer 7% GI bleed 0.7%	1 × 200 mg/day, *n *= 189	*n *= 182	Paracetamol 4 × 1,000 mg/day, *n *= 185	2 × 6 crossover	WOMAC MDHAQ Investigator global assessment Patient's assessments of helpfulness and arthritis SF-36	Withdrawals Adverse events Serious adverse events Laboratory tests	556

***Rheumatoid arthritis***									

C-012	Adult RA with flare (ACR) ≥ 6 months, requiring NSAID, FCC 1–3 Age 56 (range 21–86) years Duration of disease 11 (range 0.5–50) years 78% female 84% Caucasian	Cardioprotective ASA permitted NSAID intolerance 9% Gastroduodenal ulcer 3% GI bleed 0.6% CVD 43%	2 × 40 mg/day, *n *= 80 2 × 200 mg/day, *n *= 82 2 × 400 mg/day, *n *= 81	*n *= 84	No active comparator	4	Patient's global rating of arthritis Arthritis pain, joint tenderness, joint swelling	Withdrawals Adverse events Serious adverse events Laboratory tests	327
C-022	RA with flare (ACR) requiring NSAID, FCC 1–3 No ulcer at baseline endoscopy Age 54 (range 20–90) years Duration of disease 10 (0.3–58) years 73% female 86% Caucasian	Cardioprotective ASA permitted NSAID intolerance 10% Gastroduodenal ulcer 15% GI bleed 2% CVD 44%	2 × 100 mg/day, *n *= 240 2 × 200 mg/day, *n *= 235 2 × 400 mg/day, *n *= 217	*n *= 231	Naproxen 2 × 500 mg/day, *n *= 225	12	Patient's global assessment of arthritis Physician's global assessment of arthritic condition No. of swollen joints ACR-20 responder index No. of tender/painful joints	Withdrawals Adverse events Serious adverse events Laboratory tests Endoscopic ulcers	1,148
C-023	RA (ACR) with flare requiring NSAID, FCC 1–3 Age 55 (range 21–84) years Duration of disease 10 (range 0.3–60) years 73% female 86% Caucasian	Cardioprotective ASA permitted NSAID intolerance 10% Gastroduodenal ulcer 8% GI bleed 1% CVD 44%	2 × 100 mg/day, *n *= 228 2 × 200 mg/day, *n *= 218 2 × 400 mg/day, *n *= 217	*n *= 221	Naproxen 2 × 500 mg/day, *n *= 218	12	Patient's global assessment of arthritis Physician's global assessment of arthritic condition No. of swollen joints ACR -20 responder index No. of tender/painful joints	Withdrawals Adverse events Serious adverse events Laboratory tests	1,102
C-041	Adult onset RA (ACR) ≥ 6 months, requiring NSAID, FCC 1–3 No ulcer at baseline endoscopy Age 55 (range 20–85) years Duration of disease10 (0.6–53) years 73% female 98% Caucasian	Cardioprotective ASA not permitted NSAID intolerance 7% Gastroduodenal ulcer 8% GI bleed 0.7% CVD 25%	2 × 200 mg/day, *n *= 326	No placebo	Diclofenac (slow release) 2 × 75 mg/day, *n *= 329	24	Patient's global assessment Physician's global assessment Swollen joints Patient's assessment of arthritis pain SF-36	Withdrawals Adverse events Serious adverse events Laboratory tests Endoscopic ulcers (not all patients had endoscopy)	655

***Osteoarthritis and rheumatoid arthritis***									

C-062	OA/RA ≥ 3 months, requiring NSAID, FCC 1–3 No ulcer at baseline endoscopy Duration of OA 10 (0.3–50) years, RA 10 (0.4–43) years Age 57 (range 22–86) years 67% female 83% Caucasian	Cardioprotective ASA permitted NSAID intolerance 13% Gastroduodenal ulcer 20% GI bleed 4% CVD 53%	2 × 200 mg/day, *n *= 269	No placebo	Naproxen 2 × 500 mg/day, *n *= 267	12	Patient's global assessment Physcian's global assessment SF-36	Withdrawals Adverse events Serious adverse events Laboratory tests Endoscopic ulcers	536
C-071	OA/RA ≥ 3 months, requiring NSAID, FCC 1–3 No ulcer at baseline Age 57 (22–87) years Duration of disease 10 (0.3–48) years 68% female 82% Caucasian	Cardioprotective ASA permitted NSAID intolerance 7% Gastroduodenal ulcer 12% GI bleed 2% CVD 42%	2 × 200 mg/day, *n *= 365	No placebo	Diclofenac 2 × 75 mg/day, *n *= 387 Ibuprofen 3 × 800 mg/day, *n *= 345	12	Patient's global assessment Physcian's global assessment SF-36	Withdrawals Adverse events Serious adverse events Laboratory tests Endoscopic ulcers	1,097
C-102	OA/RA, requiring NSAID >3 months Age 60 (range 18–90) years 69% female 88% Caucasian	Cardioprotective ASA permitted NSAID intolerance 9% Gastroduodenal ulcer 8% GI bleed 2% CVD 40%	2 × 400 mg/day, *n *= 3,987	No placebo	Ibuprofen 3 × 800 mg/day, *n *= 1,985 Diclofenac 2 × 75 mg/day, *n *= 1,996	52	Patient's global assessment Patient's assessment of arthritis pain SF-36 SODA	Withdrawals Adverse events Serious adverse events Laboratory tests CSUGIEs	7,968
C-105	OA/RA (documented clinical diagnosis for ≥ 3 months), requiring NSAID, FCC 1–3 Age 50 (range 17–78) years Duration of disease not given 84% female Asian population	Cardioprotective ASA permitted Gastroduodenal ulcer 0.5% GI bleed 0.02% CVD 1%	2 × 100 mg/day, *n *= 327	No placebo	Diclofenac 2 × 50 mg/day, *n *= 330	12	Patient's global assessment Physcian's global assessment Patient's assessment of arthritis pain	Withdrawals Adverse events Serious adverse events Laboratory tests Endoscopic ulcers	657
C-106	OA/RA (documented clinical diagnosis), requiring NSAID, FCC 1–3 Age 55 (range 18–80) years Duration of disease not given 17% female ≥ 99% Asian	Cardioprotective ASA permitted Gastroduodenal ulcer 9% GI bleed 3% CVD 10%	2 × 100 mg/day, *n *= 63	No placebo	Diclofenac 2 × 50 mg/day, *n *= 61	12	Patient's global assessment Physcian's global assessment Patient's assessment of arthritis pain	Withdrawals Adverse events Serious adverse events Laboratory tests Endoscopic ulcers	124
C-107	OA/RA (documented clinical diagnosis ≥ 3 months) requiring NSAID, FCC 1–3 Age 53 (range 24–88) years Duration OA 4 (0.5–13) years, RA 6 (0.5–19) years 83% female ≥ 99% Asian	Cardioprotective ASA permitted Gastroduodenal ulcer 10% GI bleed 3% CVD 14%	2 × 100 mg/day, *n *= 44	No placebo	Diclofenac 2 × 50 mg/day, *n *= 44	12	Patient's global assessment Physcian's global assessment Patient's assessment of arthritis pain	Withdrawals Adverse events Serious adverse events Laboratory tests Endoscopic ulcers	88
C-849 (Pooled 105, 106, 107)	OA/RA		2 × 100 mg/day, *n *= 434	No placebo	Diclofenac 2 × 50 mg/day, *n *= 435	12		Endoscopic ulcers (pooled 105, 106, 107)	880

All trials had a quality score of 5/5, and a validity score of 16/16. ACR, American College of Rheumatology; ASA, acetylsalicylic acid; CHF, chronic heart failure; CSUGIE, clinically significant upper gastrointestinal event; CVD, cardiovascular disease; FCC, functional capacity class; GI, gastrointestinal; ITT, intention to treat; K-L, Kellgren-Lawrence; MDHAQ, Multidimensional Health Assessment Questionnaire; NSAID, nonsteroidal anti-inflammatory drug; OA, osteoarthritis; OASI, OA severity index; QS, quality score; RA, rheumatoid arthritis; SODA, sequential occupational dexterity index; VAS, visual analogue scale; VS, validity score; WOMAC, Western Ontario and McMaster Universities Osteoarthritis Index.

**Table 2:** Analysis of discontinuations by comparator, in studies of adverse events associated with celecoxib in arthritis

			Number of		Incidence of events (%)			
Outcome and comparisons	Celecoxib daily dose	Comparator and daily dose	Trials	Patients	Celecoxib	Comparator	Relative risk^a ^(95% CI)	NNTp^b ^or NNH^c ^(95% CI)

***All-cause discontinuation***								

Celecoxib v placebo	Any	Placebo	19	9,919	28	40	**0.64 (0.61–0.68)**^a^	**8.4 (7–10)**^b^
Celecoxib v paracetamol	Any	Paracetamol 4,000 mg	2	1,056	17	25	**0.69 (0.54–0.88)**^a^	**13 (8–35)**^b^
Celecoxib v rofecoxib	Any	Rofecoxib 25 mg	5	2,671	14	14	1.0 (0.8–1.2)	
Celecoxib (200/400) v NSAID	200–400 mg	NSAID to maximum daily	19	22,616	23	23	0.96 (0.91–1.01)	
Celecoxib (any dose) v NSAID	Any	NSAID to maximum daily	20	31,711	31	34	**0.96 (0.93–0.99)**^a^	**28 (22–40)**^b^
Celecoxib (any dose) v any active	Any	Any active comparator	26	35,302	29	32	**0.95 (0.92–0.98)**^a^	**36 (27–57)**^b^

***Lack-of-efficacy discontinuation***								

Celecoxib v placebo	Any	Placebo	19	9,914	17	28	**0.53 (0.49–0.57)**^a^	**9.0 (8–11)**^b^
Celecoxib v paracetamol	Any	Paracetamol 4,000 mg	2	1,056	7.2	11	**0.66 (0.45–0.97)**^a^	**27 (14–390)**^b^
Celecoxib v rofecoxib	Any	Rofecoxib 25 mg	5	2,671	2.2	1.5	1.5 (0.84–2.6)	
Celecoxib (200/400) v NSAID	200–400 mg	NSAID to maximum daily	19	22,613	8.0	6.3	**1.1 (1.02–1.23)**^a^	58 (42–97)^c^
Celecoxib (any dose) v NSAID	Any	NSAID to maximum daily	20	31,708	11.3	10.4	1.02 (0.96–1.1)	
Celecoxib (any dose) v any active	Any	Any active comparator	26	35,299	10.6	9.6	1.0 (0.95–1.1)	
***Adverse-event discontinuation***								
Celecoxib v placebo	Any	Placebo	19	9,914	6.6	5.5	1.2 (0.97–1.4)	
Celecoxib v paracetamol	Any	Paracetamol 4,000 mg	2	1,056	4.3	5.4	0.81 (0.47–1.4)	
Celecoxib v rofecoxib	Any	Rofecoxib 25 mg	5	2,662	6.2	6.8	0.91 (0.68–1.2)	
Celecoxib (200/400) v NSAID	200–400 mg	NSAID to maximum daily	19	22,613	8.5	9.9	**0.84 (0.77–0.92)**^a^	**74 (47–180)**^b^
Celecoxib (any dose) v NSAID	Any	NSAID to maximum daily	20	31,708	11.4	14.6	**0.86 (0.81–0.91)**^a^	**31 (25–41)**^b^
Celecoxib (any dose) v any active	Any	Any active comparator	26	35,299	10.9	13.5	**0.87 (0.82–0.92)**^a^	**38 (30–51)**^b^

***Gastrointestinal-adverse-event discontinuation***								

Celecoxib v placebo	Any	Placebo	11	5,933	2.5	2.0	1.2 (0.8–1.7)	
Celecoxib v paracetamol	Any	Paracetamol 4,000 mg	2	726	1.6	2.6	0.6 (0.2–1.6)	
Celecoxib v rofecoxib	Any	Rofecoxib 25 mg	5	2,671	2.2	2.9	0.7 (0.5–1.2)	
Celecoxib (200/400) v NSAID	200–400 mg	NSAID to maximum daily	11	18,639	4.8	6.5	**0.7 (0.6–0.8)**^a^	**58 (42–98)**^b^
Celecoxib (any dose) v NSAID	Any	NSAID to maximum daily	12	27,299	6.4	9.6	**0.75 (0.7–0.8)**^a^	**31 (26–40)**^b^
Celecoxib (any dose) v any active	Any	Any active comparator	18	30,560	6	8.7	**0.75 (0.7–0.8)**^a^	**37 (30–48)**^b^

^a^Relative risk: **bold **indicates statistically significant difference. ^b^NNTp (number-needed-to-treat to prevent one event) is indicated by **bold**. ^c^NNH (number-needed-to-treat to harm one patient). CI, confidence interval; NSAID, nonsteroidal anti-inflammatory drug.

**Table 3:** Discontinuations of treatment in arthritis because of lack of efficacy or adverse events

			Lack-of-efficacy discontinuations			Adverse-event discontinuations		
Duration (weeks)	Treatment	Dose (mg/day)	Number of events	Total number	Discontinuations, % (95% CI)	Number of events	Total number	Discontinuations, % (95% CI)

								
2–6	Placebo		339	1,925	17.6 (15.8–19.4)	97	1,925	5.0 (4.0–6.0)
	Celecoxib	<100	42	253	16.6 (12.1–21.1)	8	253	3.2 (1.0–5.4)
	Celecoxib	100	No data			No data		
	Celecoxib	200	203	4,190	4.8 (4.2–5.4)	223	4,190	5.3 (4.7–5.9)
	Celecoxib	400	12	155	7.7 (3.6–11.8)	5	155	3.2 (0.5–5.9)
	Celecoxib	800	15	180	8.3 (4.2–12.4)	14	180	7.8 (3.9–11.7)
	Paracetamol	4,000	55	502	11.0 (8.3–13.7)	27	502	5.4 (3.4–7.4)
	Rofecoxib	25	19	1,191	1.6 (0.8–2.4)	77	1,191	6.5 (5.1–7.9)
	Naproxen	1,000	5	395	1.3 (0.1–2.5)	31	395	7.8 (5.3–10.3)
	Diclofenac	100/150	13	540	2.4 (1.0–3.8)	51	540	9.4 (6.9–11.9)
								
12	Placebo		521	1,135	45.9 (43.0–48.8)	70	1,135	6.2 (4.8–7.6)
	Celecoxib	100	145	692	21 (18.1–23.9)	52	692	7.5 (5.5–9.5)
	Celecoxib	200	571	6,094	9.4 (8.6–10.2)	488	6,094	8.0 (7.4–8.6)
	Celecoxib	400	492	6,166	8.0 (7.4–8.6)	590	6,166	9.6 (8.8–10.4)
	Celecoxib	800	128	435	29.4 (25.1–33.7)	28	435	6.4 (4.0–8.8)
	Paracetamol	4,000	No data			No data		
	Rofecoxib	25	1	132	0.8 (0.0–2.4)	13	132	9.8 (4.7–14.9)
	Naproxen	1,000	374	2,399	15.6 (14.2–17.0)	316	2,399	13.2 (11.8–14.6)
	Diclofenac	100/150	120	4,311	2.8 (2.2–3.4)	338	4,311	7.8 (7.0–8.6)
	Ibuprofen	2,400	14	345	4.1 (1.9–6.3)	37	345	10.7 (7.4–14)
								
24+	Placebo		No data			No data		
	Celecoxib	100	No data			No data		
	Celecoxib	200	No data			No data		
	Celecoxib	400	26	326	8.0 (5.1–10.9)	34	326	10.4 (7.1–13.7)
	Celecoxib	800	691	3,987	17.3 (16.1–18.5)	892	3,987	22.4 (21–23.8)
	Paracetamol	4,000	No data			No data		
	Rofecoxib	25	No data			No data		
	Naproxen	1,000	No data			No data		
	Diclofenac	100/150	331	2,325	14.2 (12.8–15.6)	593	2,325	25.5 (23.7–27.3)
	Ibuprofen	2,400	456	1,985	23.0 (21.2–24.8)	456	1,985	23.0 (21.2–24.8)

CI, confidence interval.

**Table 4:** Analysis of arthritis patients according to gastrointestinal adverse events

			Number of		Incidence of events (%)			
Outcome and comparisons	Celecoxib daily dose	Comparator and daily dose	Trials	Patients	Celecoxib	Comparator	Relative risk^a ^(95% CI)	NNTp^b ^or NNH^c ^(95% CI)

***Patient with any adverse event***								

Celecoxib v placebo	Any	Placebo	19	9,919	55	48	**1.08 (1.04–1.13)**^a^	15 (11–21)^c^
Celecoxib v paracetamol	Any	Paracetamol 4,000 mg	2	1,056	32	32	1.0 (0.84–1.2)	
Celecoxib v rofecoxib	Any	Rofecoxib 25 mg	3	769	48	49	0.97 (0.84–1.1)	
Celecoxib (200/400) v NSAID	200–400 mg	NSAID to maximum daily	19	22,615	45	50	**0.92 (0.89–0.95)**^a^	**18 (14–23)**^b^
Celecoxib (any dose) v NSAID	Any	NSAID to maximum daily	20	31,711	53	60	**0.96 (0.94–0.98)**^a^	**15 (13–18)**^b^
Celecoxib (any dose) v any active	Any	Any active comparator	24	33,400	53	59	**0.96 (0.94–0.98)**^a^	**17 (14–21)**^b^

***Patient with any treatment-related adverse event***								

Celecoxib v placebo	Any	Placebo	19	9,919	9.5	8.1	**1.22 (1.06–1.40)**^a^	71 (39–450)^c^
Celecoxib v paracetamol	Any	Paracetamol 4,000 mg	3	1,056	9.0	8.8	1.04 (0.71–1.5)	
Celecoxib v rofecoxib	Any	Rofecoxib 25 mg	4	1,579	6.6	9.0	0.74 (0.53–1.04)	
Celecoxib (200/400) v NSAID	200–400 mg	NSAID to maximum daily	19	22,615	13.0	17.3	**0.77 (0.72–0.82)**^a^	**24 (19–31)**^b^
Celecoxib (any dose) v NSAID	Any	NSAID to maximum daily	19	23,743	12.7	17.3	**0.77 (0.72–0.82)**^a^	**22 (18–27)**^b^
Celecoxib (any dose) v any active	Any	Any active comparator	24	26,242	12.3	16.2	**0.78 (0.73–0.83)**^a^	**26 (21–33)**^b^

***Patient with any serious adverse event***								

Celecoxib v placebo	Any	Placebo	19	9,919	1.0	1.4	**0.67 (0.46–0.98)**^a^	**280 (120–790)**^b^
Celecoxib v paracetamol	Any	Paracetamol 4,000 mg	2	1,056	0.5	0.6	0.76 (0.14–4.1)	
Celecoxib v rofecoxib	Any	Rofecoxib 25 mg	5	2,671	2.3	2.1	1.1 (0.68–1.9)	
Celecoxib (200/400) v NSAID	200–400 mg	NSAID to maximum daily	19	22,612	2.5	2.6	0.91 (0.77–1.08)	
Celecoxib (any dose) v NSAID	Any	NSAID to maximum daily	20	31,708	3.3	3.6	1.02 (0.91–1.15)	
Celecoxib (any dose) v any active	Any	Any active comparator	26	35,299	3.2	3.4	1.02 (0.91–1.15)	

***Patient with any gastrointestinal adverse event***								

Celecoxib v placebo	Any	Placebo	17	9,512	26.0	19.0	**1.2 (1.1–1.4)**^a^	14 (12–19)^c^
Celecoxib v paracetamol	Any	Paracetamol 4,000 mg	2	1,056	12.0	11.0	1.1 (0.8–1.6)	
Celecoxib v rofecoxib	Any	Rofecoxib 25 mg	5	2,671	16.0	18.0	0.87 (0.74–1.03)	
Celecoxib (200/400) v NSAID	200–400 mg	NSAID to maximum daily	18	30,043	26.0	34.0	**0.84 (0.81–0.87)**^a^	**12 (10–13)**^b^
Celecoxib (any dose) v NSAID	Any	NSAID to maximum daily	18	31,171	26.0	34.0	**0.84 (0.81–0.87)**^a^	**12 (10–13)**^b^
Celecoxib (any dose) v any active	Any	Any active comparator	24	34,762	26.0	32.0	**0.85 (0.82–0.88)**^a^	**14 (12–16)**^b^

^a^Relative risk: **bold **indicates statistically significant difference. ^b^NNTp (number-needed-to-treat to prevent one event) is indicated by **bold**. ^c^NNH (number-needed-to-treat to harm one patient). CI, confidence interval; NSAID, nonsteroidal anti-inflammatory drug.

**Table 5a:** Gastrointestinal adverse events reported in studies of arthritis patients (part 1)

			Number of		Incidence of events (%)			
Outcome and comparisons	Celecoxib daily dose	Comparator and daily dose	Trials	Patients	Celecoxib	Comparator	Relative risk^a ^(95% CI)	NNTp^b ^or NNH^c ^(95% CI)

***Gastrointestinal tolerability***								

Celecoxib v placebo	Any	Placebo	19	9,919	5.3	4.6	1.0 (0.82–1.2)	
Celecoxib v paracetamol	Any	Paracetamol 4,000 mg	2	1,056	2.0	2.0	1.0 (0.43–2.4)	
Celecoxib v rofecoxib	Any	Rofecoxib 25 mg	5	2,671	3.2	4.4	0.72 (0.49–1.06)	
Celecoxib (200/400) v NSAID	200–400 mg	NSAID to maximum daily	19	22,615	5.4	8.9	**0.62 (0.56–0.68)**	**28 (24–36)**^b^
Celecoxib (any dose) v NSAID	Any	NSAID to maximum daily	19	23,743	5.5	8.9	**0.61 (0.55–0.67)**	**29 (24–36)**^b^
Celecoxib (any dose) v any active	Any	Any active comparator	25	27,334	5.2	8.0	**0.63 (0.57–0.69)**	**35 (29–45)**^b^

***Nausea***								

Celecoxib v placebo	Any	Placebo	17	9,510	2.7	3.4	**0.76 (0.60–0.97)**	**155 (71–840)**^b^
Celecoxib v paracetamol	Any	Paracetamol 4,000 mg	2	1,056	2.9	1.8	1.6 (0.73–3.7)	
Celecoxib v rofecoxib	Any	Rofecoxib 25 mg	4	1,579	1.8	2.8	0.62 (0.32–1.2)	
Celecoxib (200/400) v NSAID	200–400 mg	NSAID to maximum daily	17	22,072	2.7	3.3	0.87 (0.74–1.02)	
Celecoxib (any dose) v NSAID	Any	NSAID to maximum daily	18	31,168	3.8	5.6	**0.80 (0.72–0.89)**	**56 (44–77)**^b^
Celecoxib (any dose) v any active	Any	Any active comparator	23	33,667	3.7	5.3	**0.81 (0.73–0.90)**	**63 (49–88)**^b^

***Vomiting***								

Celecoxib v placebo	Any	Placebo	15	9,030	1.1	0.7	1.4 (0.86–2.4)	
Celecoxib v paracetamol	Any	Paracetamol 4,000 mg	2	1,056	0.7	1.0	0.73 (0.19–2.7)	
Celecoxib v rofecoxib	Any	Rofecoxib 25 mg	3	769	1.0	0.8	1.3 (0.29-5.7)	
Celecoxib (200/400) v NSAID	200–400 mg	NSAID to maximum daily	16	21,825	0.8	1.4	**0.64 (0.49–0.83)**	**173 (115–350)**^b^
Celecoxib (any dose) v NSAID	Any	NSAID to maximum daily	17	30,921	1.2	1.9	**0.75 (0.62–0.90)**	**144 (100–250)**^b^
Celecoxib (any dose) v any active	Any	Any active comparator	21	32,610	1.2	1.9	**0.76 (0.64–0.91)**	**156 (110–280)**^b^

^a^Relative risk: **bold **indicates statistically significant difference. ^b^NNTp (number-needed-to-treat to prevent one event) is indicated by **bold**. ^c^NNH (number-needed-to-treat to harm one patient). CI, confidence interval; NSAID, nonsteroidal anti-inflammatory drug.

**Table 5b:** Gastrointestinal adverse events reported in studies of arthritis patients (part 2)

			Number of		Incidence of events (%)			
Outcome/ comparisons	Celecoxib daily dose	Comparator and daily dose	Trials	Patients	Celecoxib	Comparator	Relative risk^a ^(95% CI)	NNTp^b ^or NNH^c ^(95% CI)

***Abdominal pain***								

Celecoxib v placebo	Any	Placebo	19	9,919	3.6	2.9	1.2 (0.92–1.5)	
Celecoxib v paracetamol	Any	Paracetamol 4,000 mg	2	1,056	0.9	2.0	0.45 (0.15–1.3)	
Celecoxib v rofecoxib	Any	Rofecoxib 25 mg	5	2,671	2.7	4.2	**0.64 (0.42–0.97)**^a^	**67 (35–920)**^b^
Celecoxib (200/400) v NSAID	200–400 mg	NSAID to maximum daily	19	22,615	5.3	7.8	**0.75 (0.68–0.83)**^a^	**41 (32–57)**^b^
Celecoxib (any dose) v NSAID	Any	NSAID to maximum daily	20	31,711	6.6	10.0	**0.76 (0.70–0.82)**^a^	**29 (25–36)**^b^
Celecoxib (any dose) v any active	Any	Any active comparator	26	35,302	6.2	9.2	**0.75 (0.70–0.81)**^a^	**33 (28–41)**^b^

***Dyspepsia***								

Celecoxib v placebo	Any	Placebo	19	9,919	6.9	4.8	**1.30 (1.08–1.6)**^a^	46 (32–84)^c^
Celecoxib v paracetamol	Any	Paracetamol 4,000 mg	2	1,056	2.9	2.2	1.34 (0.63–2.9)	
Celecoxib v rofecoxib	Any	Rofecoxib 25 mg	5	2,671	4.4	4.9	0.89 (0.63–1.3)	
Celecoxib (200/400) v NSAID	200–400 mg	NSAID to maximum daily	19	22,615	5.7	7.3	**0.79 (0.71–0.88)**^a^	**61 (43–100)**^b^
Celecoxib (any dose) v NSAID	Any	NSAID to maximum daily	20	31,711	8.1	10.7	**0.84 (0.78–0.90)**^a^	**39 (31–52)**^b^
Celecoxib (any dose) v any active	Any	Any active comparator	26	35,302	7.8	9.9	**0.85 (0.79–0.91)**^a^	**48 (37–68)**^b^

***Diarrhoea***								

Celecoxib v placebo	Any	Placebo	17	9,510	5.1	3.5	**1.45 (1.16–1.82)**^a^	53 (37–97)^c^
Celecoxib v paracetamol	Any	Paracetamol 4,000 mg	2	1,056	2.2	4.6	**0.48 (0.24–0.95)**^a^	**41 (22–450)**^b^
Celecoxib v rofecoxib	Any	Rofecoxib 25 mg	5	2,671	4.1	4.4	0.93 (0.65–1.3)	
Celecoxib (200/400) v NSAID	200–400 mg	NSAID to maximum daily	17	22,071	4.3	4.9	0.96 (0.85–1.1)	
Celecoxib (any dose) v NSAID	Any	NSAID to maximum daily	18	31,167	5.8	6.9	0.96 (0.88–1.1)	
Celecoxib (any dose) v any active	Any	Any active comparator	24	34,758	5.6	6.6	0.95 (0.87–1.03)	

***Clinical ulcers and bleeds***								

Celecoxib v placebo	Any	Placebo	16	9,321	0.03	0.05		3 events
Celecoxib v paracetamol	Any	Paracetamol 4,000 mg	2	1,056	0.0	0.0		0 events
Celecoxib v rofecoxib	Any	Rofecoxib 25 mg	3	897	0.3	0.0		1 event
Celecoxib (200/400) v NSAID	200–400 mg	NSAID to maximum daily	17	22,075	0.2	0.6	**0.35 (0.22–0.56)**^a^	**250 (170–450)**^b^
Celecoxib (any dose) v NSAID	Any	NSAID to maximum daily	18	31,171	0.4	0.9	**0.61 (0.46–0.81)**^a^	**200 (140–320)**^b^
Celecoxib (any dose) v any active	Any	Any active comparator	22	32,508	0.4	0.8	**0.61 (0.46–0.81)**^a^	**210 (150–350)**^b^

^a^Relative risk: **bold **indicates statistically significant difference. ^b^NNTp (number-needed-to-treat to prevent one event) is indicated by **bold**. ^c^NNH (number-needed-to-treat to harm one patient). CI, confidence interval; NSAID, nonsteroidal anti-inflammatory drug.

**Table 6:** Cardio-renal adverse events reported in studies of patients treated for arthritis

			Number of		Incidence of events (%)			
Outcome/ comparisons	Celecoxib daily dose	Comparator and daily dose	Trials	Patients	Celecoxib	Comparator	Relative risk^a ^(95% CI)	NNTp^b ^or NNH^c ^(95% CI)

***Myocardial infarction***								
Celecoxib v placebo	Any	Placebo	16	9,315	0.12	0.07		10 events
Celecoxib v paracetamol	Any	Paracetamol 4,000 mg	2	1,056	0.00	0.00		0 events
Celecoxib v rofecoxib	Any	Rofecoxib 25 mg	5	2,667	0.00	0.08		1 event
Celecoxib (200/400) v NSAID	200–400 mg	NSAID to maximum daily	16	21,818	0.15	0.04	1.9 (0.87–4.1)	23 events
Celecoxib (any dose) v NSAID	Any	NSAID to maximum daily	17	30,220	0.22	0.14	1.6 (0.93–2.6)	56 events
Celecoxib (any dose) v any active	Any	Any active comparator	23	34,174	0.19	0.13	1.4 (0.87–2.3)	57 events
Celecoxib (any dose) v any comparator	Any	Any comparator	30	38,499	0.18	0.12	1.4 (0.88–2.2)	59 events
Celecoxib (any dose) v noncoxib comparator	Any	Any noncoxib comparator	28	36,316	0.19	0.12	1.4 (0.88–2.2)	57 events

***Cardiac failure***								

Celecoxib v placebo	Any	Placebo	16	9,834	0.06	0.03		5 events
Celecoxib v paracetamol	Any	Paracetamol 4,000 mg	2	1,056	0.00	0.00		0 events
Celecoxib v rofecoxib	Any	Rofecoxib 25 mg	5	2,671	0.15	0.60		10 events
Celecoxib (200/400) v NSAID	200–400 mg	NSAID to maximum daily	15	21,859	0.06	0.15	0.54 (0.29–1.02)	21 events
Celecoxib (any dose) v NSAID	Any	NSAID to maximum daily	17	30,917	0.11	0.20	0.70 (0.43–1.1)	45 events
Celecoxib (any dose) v any active	Any	Any active comparator	23	34,512	0.11	0.23	0.64 (0.41–1.0)	55 events

***Raised creatinine (above 1.3 × upper limit of normal)***								

Celecoxib v placebo	Any	Placebo	5	2,776	1.3	0.7	1.65 (0.69–4.0)	
Celecoxib v paracetamol	Any	Paracetamol 4,000 mg						
Celecoxib v rofecoxib	Any	Rofecoxib 25 mg						
Celecoxib (200/400) v NSAID	200–400 mg	NSAID to maximum daily	9	15,319	0.3	0.5	0.78 (0.46–1.3)	
Celecoxib (any dose) v NSAID	Any	NSAID to maximum daily						
Celecoxib (any dose) v any active	Any	Any active comparator	10	15,657	0.3	0.5	0.79 (0.47–1.3)	

***Hypertension and aggravated hypertension***								

Celecoxib v placebo	Any	Placebo	16	9,321	1.0	0.6	1.4 (0.85–2.4)	
Celecoxib v paracetamol	Any	Paracetamol 4,000 mg	2	1,056	0.2	0.6		4 events
Celecoxib v rofecoxib	Any	Rofecoxib 25 mg	5	2,671	3.5	4.6	0.75 (0.52–1.1)	
Celecoxib (200/400) v NSAID	200–400 mg	NSAID to maximum daily	16	22,518	1.3	1.4	0.92 (0.73–1.2)	
Celecoxib (any dose) v NSAID	Any	NSAID to maximum daily	17	30,921	1.6	1.6	1.1 (0.90–1.3)	
Celecoxib (any dose) v any active	Any	Any active comparator	23	34,512	1.7	1.8	1.0 (0.86–1.2)	

***Oedema at any site***								

Celecoxib v placebo	Any	Placebo	16	9,321	2.6	1.4	**1.9 (1.4–2.7)**	**79 (54–145)**^c^
Celecoxib v paracetamol	Any	Paracetamol 4,000 mg	2	1,056	2.3	1.8	1.3 (0.56–3.0)	
Celecoxib v rofecoxib	Any	Rofecoxib 25 mg	5	2,671	18.0	25.0	**0.72 (0.62–0.83)**	**14 (10–25)**^b^
Celecoxib (200/400) v NSAID	200–400 mg	NSAID to maximum daily	16	21,825	2.4	2.5	0.98 (0.82–1.2)	
Celecoxib (any dose) v NSAID	Any	NSAID to maximum daily	17	30,921	2.9	3.5	0.92 (0.81–1.05)	
Celecoxib (any dose) v any active	Any	Any active comparator	23	34,512	3.8	5.4	**0.84 (0.76–0.92)**	**62 (48–87)**^b^

^a^Relative risk: **bold **indicates statistically significant difference. ^b^NNTp (number-needed-to-treat to prevent one event) is indicated by **bold**. ^c^NNH (number-needed-to-treat to harm one patient; CI, confidence interval; NSAID, nonsteroidal anti-inflammatory drug.

**Table 7:** Analysis of changes to haematological parameters in patients treated for arthritis

			Number of		Incidence of events (%)			
Outcome/ comparisons	Celecoxib daily dose	Comparator and daily dose	Trials	Patients	Celecoxib	Comparator	Relative risk^a ^(95% CI)	NNTp^b ^or NNH^c ^(95% CI)

***Haemoglobin fall of 20 g/L or more***								

Celecoxib v placebo	Any	Placebo	5	3,577	0.8	0.5	1.5 (0.56–4.0)	
Celecoxib v paracetamol	Any	Paracetamol 4,000 mg						
Celecoxib v rofecoxib	Any	Rofecoxib 25 mg						
Celecoxib (200/400) v NSAID	200–400 mg	NSAID to maximum daily	10	15,746	1.1	2.2	**0.71 (0.55–0.91)**^a^	**92 (66–150)**^b^
Celecoxib (any dose) v NSAID	Any	NSAID to maximum daily	10	16,180	1.1	2.2	**0.72 (0.56–0.92)**^a^	**93 (67–150)**^b^
Celecoxib (any dose) v any active	Any	Any active comparator	11	16,990	1.1	2.1	**0.72 (0.56–0.92)**^a^	**100 (71–170)**^b^

***Haematocrit fall of 5% or more***								

Celecoxib v placebo	Any	Placebo	9	6,442	8.1	6.5	1.20 (0.98–1.5)	
Celecoxib v paracetamol	Any	Paracetamol 4,000 mg						
Celecoxib v rofecoxib	Any	Rofecoxib 25 mg	2	962	12.6	17.1	0.74 (0.54–1.01)	
Celecoxib (200/400) v NSAID	200–400 mg	NSAID to maximum daily	12	6,910	9.9	15.4	**0.77 (0.68–0.88)**^a^	**18 (14–25)**^b^
Celecoxib (any dose) v NSAID	Any	NSAID to maximum daily	12	8,038	9.9	15.4	**0.78 (0.69–0.89)**^a^	**18 (14–25)**^b^
Celecoxib (any dose) v any active	Any	Any active comparator	14	8,970	10.1	15.6	**0.78 (0.69–0.88)**^a^	**18 (14–25)**^b^

^a^Relative risk: **bold **indicates statistically significant difference. ^b^NNTp (number-needed-to-treat to prevent one event) is indicated by **bold**. ^c^NNH (number-needed-to-treat to harm one patient). CI, confidence interval; NSAID, nonsteroidal anti-inflammatory drug.

**Table 8:** Endoscopically detected ulcers in patients treated for arthritis, with and without aspirin

			Number of		Incidence of events (%)			
Outcome/ comparisons	Celecoxib daily dose	Comparator and daily dose	Trials	Patients	Celecoxib	Comparator	Relative risk^a ^(95% CI)	NNTp^b ^or NNH^c ^(95% CI)

***Analysis irrespective of aspirin use***								

Celecoxib v placebo	Any	Placebo	2	1,737	3.9	2.2	1.8 (0.89–3.6)	
Celecoxib (200/400) v NSAID	200–400 mg	NSAID to maximum daily	6	4,135	4.6	16.3	**0.30 (0.24–0.37)**^a^	**8.6 (7.4–10)**^b^
Celecoxib (any dose) v NSAID	Any	NSAID to maximum daily	6	4,565	4.5	16.3	**0.29 (0.24–0.36)**^a^	**8.4 (7.3–10)**^b^

***Analysis without aspirin use***								

Celecoxib v placebo	Any	Placebo	2	1,537	3.3	1.9	1.8 (0.79–3.9)	
Celecoxib (200/400) v NSAID	200–400 mg	NSAID to maximum daily	5	3,053	4.5	17.6	**0.28 (0.22–0.36)**^a^	**7.6 (6.5–9.1)**^b^
Celecoxib (any dose) v NSAID	Any	NSAID to maximum daily	5	3,440	4.2	17.6	**0.28 (0.22–0.36)**^a^	**7.5 (6.4–8.9)**^b^

***Analysis with aspirin use***								

Celecoxib v placebo	Any	Placebo	2	200	7.9	4.1	1.7 (0.45–6.3)	
Celecoxib (200/400) v NSAID	200–400 mg	NSAID to maximum daily	5	344	10.0	23.8	**0.47 (0.27–0.83)**^a^	**7.3 (4.6–17)**^b^
Celecoxib (any dose) v NSAID	Any	NSAID to maximum daily	5	387	9.9	23.8	**0.48 (0.28–0.83)**^a^	**7.2 (4.7–16)**^b^

^a^Relative risk: **bold **indicates statistically significant difference. ^b^NNTp (number-needed-to-treat to prevent one event) is indicated by **bold**. ^c^NNH (number-needed-to-treat to harm one patient). CI, confidence interval; NSAID, nonsteroidal anti-inflammatory drug.
