# Supplementary material for: Tolerability and adverse events in clinical trials of celecoxib in osteoarthritis and rheumatoid arthritis: systematic review and meta-analysis of information from company clinical trial reports
Source: Arthritis Res Ther. 2005 Mar 24;7(3):R644–65. doi: 10.1186/ar1704 (PMC1174947; doi:10.1186/ar1704)
Supplement: Additional File 1 — A Word file containing a table giving trial inclusion and exclusion criteria for arthritis. [file ar1704-S1.doc]

### Additional file 1: Trial inclusion and exclusion criteria relating to arthritis

#### Osteoarthritis

Inclusion criteria for trials enrolling patients with osteoarthritis (OA) included, but were not confined to:

- Patients with symptomatic OA as defined by the American College of Rheumatology (ACR) criteria or modified ACR criteria
- Baseline score of at least 40 mm on visual analogue scale
- Baseline functional capacity class 1 to 3, in which class 1 was complete ability to carry out usual duties without handicap, and class 3 was limited ability to carry out duties of usual occupation or self-care. This classification excluded largely or wholly incapacitated persons.
- Patient and physician global assessment of arthritis rating of poor or very poor

Trial exclusion criteria for patients with OA included, but were not confined to:

- Inflammatory arthritis, gout, pseudogout, Paget’s disease
- Patients suffering from chronic pain syndromes that may interfere with the index joint
- Severe bursitis (knee studies only)
- Symptomatic trochanteric bursitis (hip studies only)
- Acute joint trauma
- Complete loss of articular cartilage
- Intra-articular/intramuscular steroids in previous 4 weeks
- Patients with active gastrointestinal (GI) disease
- GI tract ulceration within 30 days before the first dose of trial medication
- Significant bleeding disorder
- History of gastric or duodenal surgery

#### Rheumatoid arthritis

Inclusion criteria for trials enrolling patients with rheumatoid arthritis included, but were not confined to:

- Adult onset RA, as defined by the ACR, of at least three months’ duration
- RA stable on traditional nonsteroidal anti-inflammatory drus (NSAID) therapy for at least one month
- Baseline functional capacity class 1 to 3
- Patients with RA in ‘flare state’ at baseline or expected to benefit from an NSAID or change of NSAID

Trial exclusion criteria for patients with rheumatoid arthritis included, but were not confined to:

- Any other form of inflammatory arthritis
- Any secondary/noninflammatory arthritis that may interfere with evaluation
- Active GI disease
- Significant coagulation disorders
- GI ulceration in previous 30 days
- Intra-articular/intramuscular steroids in previous 4 weeks
- New treatment with gold/antimalarials in previous 3 months.
- New treatment or altered dosing regimes with methotrexate >25mg/week, sulphasalazine >3 g/day, azathioprine, penicillamine, etanercept, leflunomide in previous 8 to 12 weeks. Patients established on disease-modifying antirheumatic drugs were allowed into the studies.
